# Combination of PARP inhibitor olaparib and a vascular disrupting agent reduces tumor growth in a preclinical model of prostate cancer

**DOI:** 10.1007/s00280-026-04946-1

**Published:** 2026-09-17

**Authors:** Evangelia Sereti, Susan Evans-Axelsson, Jan Törnell, Stefan Rehnmark, Marita Högberg, Anders Bjartell

**Affiliations:** 1 Department of Translational Medicine, Division of Urological Cancers, Lund University, Jan Waldenströms gata 14, Malmö, 20502 Sweden; 2 Innoext AB, Göteborg, Sweden; 3 LIDDS Pharma AB, Göteborg, Sweden; 4 Department of Urology, Skåne University Hospital, Malmö, Sweden

**Keywords:** Metastatic castration resistant prostate cancer, NOV202, olaparib, PARPi, Preclinical animal models

## Abstract

**Purpose:**

This study aims to characterize the antiproliferative and antitumor activity of NOV202, a novel vascular-disrupting and microtubule-destabilizing compound, alone and in combination withthe PARP inhibitor olaparib, in preclinical prostate cancer models in vitro and in vivo.

**Methods:**

DU145 (carrying a BRCA2 variant of unknown significance) and PC-3 (BRCA2 wildtype) prostate cancer cells were implanted in NMRI nude mice. Mice received oral olaparib, NOV202, or both for 21 days. Tumor growth was monitored via bioluminescence imaging and caliper measurements. By immunohistochemistry we assessed DNA damage (γH2AX), homologous recombination repair (RAD51), hypoxia (HIF-1α), proliferation (Ki-67), vascularization (CD31) and apoptosis (cleaved caspase 3). Cell viability assays and western blot analyses were performed to assess treatment-induced DNA-damage and apoptosis markers and to evaluate synergistic effects in vitro.

**Results:**

Olaparib and NOV202 independently reduced tumor growth in both xenograft models. NOV202 was the dominant single agent in vivo, and adding olaparib resulted in a modest increase in tumor growth inhibition over NOV202 alone. Immunohistochemistry showed treatment-associated increases in DNA damage and reduced vascularization in tumors. All treatments were well tolerated with minimal signs of toxicity. In vitro, the combination induced synergistic antiproliferative effects. At the protein level, DNA-damage and apoptosis markers were increased mainly by olaparib and were not further enhanced by the combination.

**Conclusion:**

NOV202 showed potent single-agent antitumor activity in both prostate cancer xenograft models. The combination with olaparib was synergistic in vitro at low nanomolar NOV202 concentrations but did not provide a clear additional benefit over NOV202 alone in vivo.

**Supplementary Information:**

The online version contains supplementary material available at https://doi.org/10.1007/s00280-026-04946-1.

## Introduction

Prostate cancer (PCa) is the second most frequently diagnosed cancer in men and a leading cause of cancer-related death, worldwide [1]. Μetastatic castration-resistant prostate cancer (mCRPC) is an aggressive and heterogenous stage of the disease, often associated with poor clinical outcomes [2] and acquired resistance to various therapies. Genomic profiling has revealed that homologous recombination repair (HRR) gene alterations are present in 10–25% of mCRPC patients and the most commonly mutated HRR gene in this population is *BRCA2* (44%) [3]. Clinical trials have shown that mCRPC patients with HRR deficiencies responded favorably to PARPi [4–6].

PARP inhibitors, previously established in the treatment of ovarian, pancreatic and breast cancers [7–9], have recently gained approval for the use in PCa. Three PARPi, olaparib, rucaparib and talazoparib have received regulatory approval for patients with mCRPC and specific HRR alterations [10–12]. While these trials have demonstrated the clinical benefit of PARPi in HRR-mutated mCRPC, the level of benefit varies among patients with different HRR alterations [13]. Clinical trials are currently evaluating patients benefit regardless of the HRR mutation status, combining PARPi with other agents [14–17] investigating the potential to extend the therapeutic benefit to HRR-proficient tumors. However, further data are needed to assess the potential benefits of combining PARPi with other agents outside the HRR-deficient subset of PCa patients.

Additionally, a considerable proportion of patients eventually develop resistance to PARPi [18, 19]. To address PARPi resistance, rational drug combinations are necessary, and research is currently focused on exploring novel anticancer agents that could enhance the therapeutic efficacy of PARPi or overcome acquired resistance improving clinical outcomes for patients.

NOV202 is a novel microtubule destabilizer agent (MDA) and a vascular disrupting agent (VDA) that has shown significant anti-proliferative effects in ovarian cancer xenografts and across a large panel of cancer cell lines [20]. VDAs induce selective blood flow disruption and subsequent tumor hypoxia [21] while MDAs disrupt microtubule formation, leading to cell-cycle arrest and apoptosis in cancer cells [22].

This study investigates the combinational efficacy of the PARP inhibitor olaparib with the novel anticancer agent NOV202. We evaluate the antiproliferative effects of olaparib, NOV202 and their combination in vitro, and further assess whether NOV202 can enhance the in vivo antitumor efficacy of olaparib in prostate cancer xenografts.

## Materials and methods

### Cell lines and reagents

DU145 (RRID: CVCL_0105) and PC-3 (RRID: CVCL_0035) cells were purchased from the American Type Culture Collection (ATCC, Manassas, VA, USA), DU145-Luciferase (DU145-Luc, RRID: CVCL_J251) cells from Anthem Biosciences (Bangalore, India) and PC-3-Luciferase (PC-3-Luc, RRID: CVCL_J265) cells from Perkin Elmer (Waltham, Massachusetts, US). All cell lines were cultured in RPMI-1640 medium and cell cultures were maintained as previously described [23]. All cell lines were authenticated using short tandem repeat (STR) profiling within the last three years. All experiments were performed with mycoplasma-free cells. NOV202 (MW 413) was provided by LIDDS AB (Göteborg, Sweden). Olaparib (HY-10162/CS-0075) was purchased by MedChemExpress. For the in vitro experiments compounds were dissolved into dimethyl sulfoxide (DMSO) at a maximum concentration of 0.1% of the total volume.

### Cell viability assay

Cells were seeded into a flat bottom 96-well plate at a density of 5000 cells per well (PC-3) or 2000 cells per well (DU145), 24 h before treatment. Compounds were serially diluted in culture medium to acquire the desired concentrations. After 72 h of treatment the cytotoxic effect of the compounds was determined by sulforhodamine B (SRB) colorimetric assay as previously described [24].

### Genetic analysis

DNA was extracted from PC-3 and DU145 cell lines using AllPrep DNA/RNA kit (Qiagen), with 250ng of DNA serving as input for the library preparation (KAPA HyperPlus, Roche Diagnostics AB). The DNA library was enriched though hybridization enrichment, followed by DNA sequencing on the NextSeq500 platform (Illumina; Kit: NSQ 500/550, Hi Output KT v2.5, 300 CYS). The analysis included demultiplexing (Picard 2.20.8), alignment (Novoalign, novocraft V4.0), variant calling (Unified Genotyper, GenomeAnalysisToolKit 3.8.1.0 and FreeBayes 1.1.0), and coverage analysis (with a threshold of 100x for each bp in coding exons and 20 bp into the introns).

### Western blot

Cell lysis and protein extraction were performed as previously described [25]. Membranes were developed with Immobilon Forte Western HRP Substrate (Cat. WBLUF0100; Millipore) and detected with Fujifilm LAS 3000 Imager. The following antibodies were used: γH_2_A_X_ (phospho S139) (ab11174; Abcam), cleaved PARP (Asp214) (5625; Cell Signaling), p-ATM (Ser1981) (13050; Cell Signaling), beta-actin (A5441; Sigma).

### Animal experiments

Prostate cancer xenografts were developed from luciferase-transfected PC-3 and DU145 cell lines. Male NMRI nude mice were inoculated subcutaneously into the lower right flank with the cells and once tumor volumes reached approximately 100mm^3^, animals were randomized into 4 groups. Mice were treated orally with vehicle, olaparib, NOV202, or their combination. Olaparib was administered once daily for 21 consecutive days (qDx21), whereas NOV202 was administered once daily for 5 consecutive days each week over 3 weeks (q5Dx3). These schedules were chosen according to the established tolerability and pharmacokinetic profiles of each agent: continuous once-daily oral dosing is the standard preclinical regimen for olaparib, whereas the intermittent 5-days-on/2-days-off schedule for NOV202 follows the regimen used in previous in vivo studies of this compound. All groups were treated over the same overall 3-week period. Mice in the vehicle group received both vehicle solutions, the olaparib vehicle [10% DMSO in 10% 2-HPβCD in PBS] and the NOV202 vehicle (70% PEG400, 0.25% Tween-80 in water), administered on the same schedules as the corresponding treatments so that every group received an equivalent vehicle exposure. Tumor size was measured with calipers. Tumor growth and response to treatment were monitored using non-invasive bioluminescence optical imaging (BLI) with the IVIS Lumina II imaging system (PerkinElmer, Hopkinton). Bioluminescence data were quantified using Living Imaging software 4.2 (Xenogen Corp., Alameda). At the experiment’s endpoint, the animals were euthanized, tumors were removed, weighted individually, fixed in 10% formalin, and embedded in paraffin for immunohistochemical analysis.

### Immunohistochemistry

Tissue sections (4 μm) from the xenograft tumors were stained using the EnVision Flex kit (K8010 DAKO) in an Autostainer Link 48 system (Dako/Agilent Technologies). The following antibodies were used: γH_2_A_X_ (phospho S139) (ab11174; Abcam), RAD51 (ab133534; Abcam), HIF-1α (MA1-516; Thermo Fisher Scientific), CD31 (77699; Cell Signaling), Ki-67 (Clone MIB-1; DAKO), Cleaved Caspase-3 (9661; Cell Signaling). Stained slides were scanned and digitized using the Aperio ScanScope CS Slide Scanner (Leica Biosystems). The area stained by each antibody was quantified by HALO image analysis software (Indica Labs, v2.3.2089.69).

### Statistical analyses

Data were analysed using GraphPad Prism 10. Statistical significance was assessed using analysis of variance (ANOVA). Dose-response curves were generated using the nonlinear regression curve fit method (4 parameters). Total growth inhibition (TGI) was calculated in GraphPad Prism. TGI at the study endpoint was calculated as TGI (%) = (1 − T/C) × 100, where T and C are the mean tumor volume (or bioluminescence signal) of the treated and vehicle-control groups, respectively. Half maximal inhibitory concentration (IC50) was calculated from dose-response curves. Combination index (CI) was calculated by CompuSyn software (CombuSyn, Inc.) using the Chou-Talalay method at non-constant ratios. Results were considered statistically significant if *P* ≤ 0.05 (*), *P* < 0.01 (**). For in vivo and in vitro experiments, the number of technical and biological replicates is indicated in the corresponding figure legends.

### Statement of animal rights

All animal experiments were conducted according to the National Research Council’s Guide for the Care and Use of Laboratory Animals with approval by the Malmö-Lund Ethical Committee at Lund University for the care and use of laboratory animals (Dnr 5.8.18-Final 14084/2019).

## Results

### Olaparib and NOV202 combination synergistically inhibits prostate cancer cell viability and growth

**Fig. 1:**
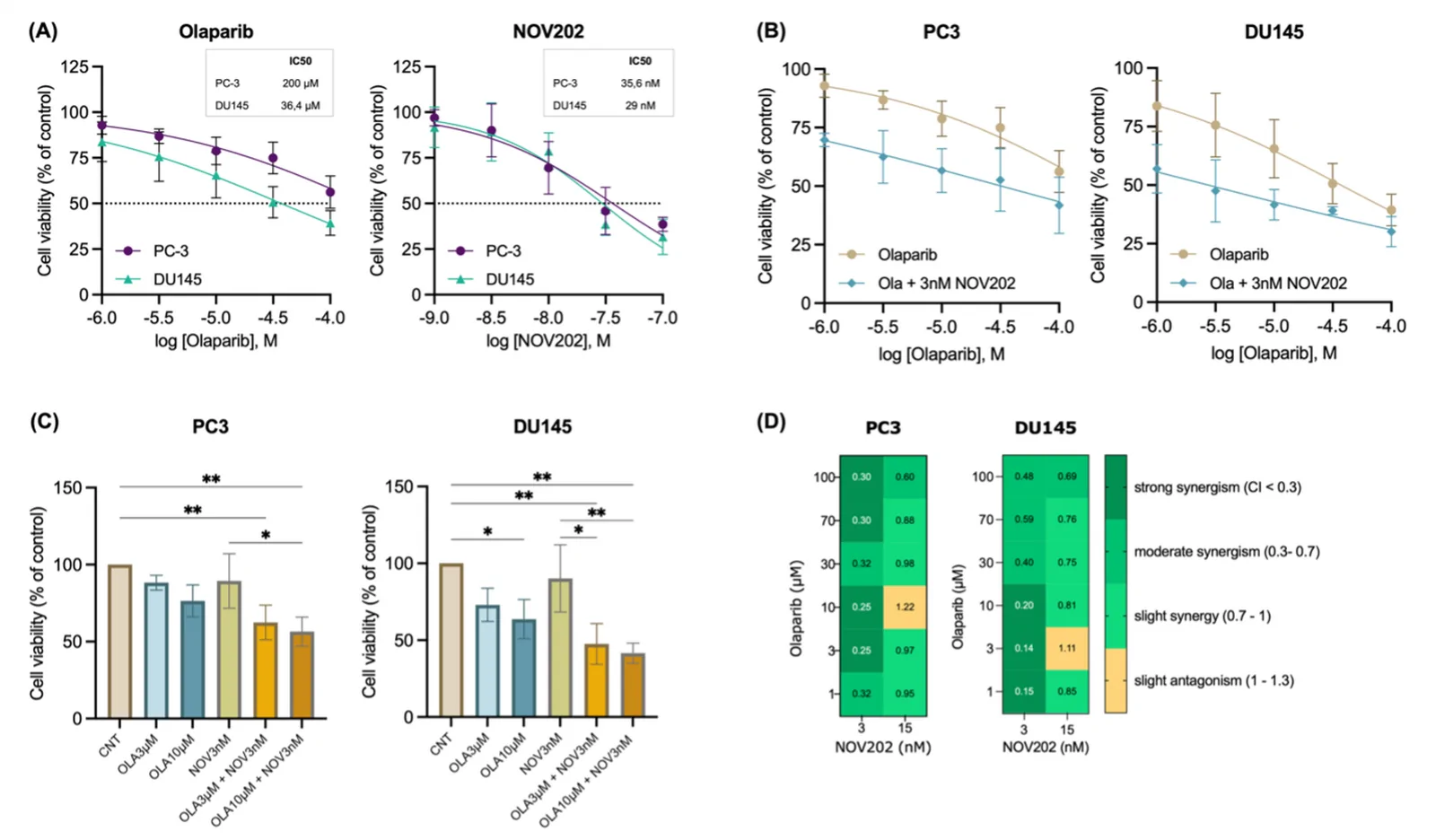
(**A**) Cell viability of PC-3 and DU145 cells treated with the indicated concentrations of olaparib and NOV202. Cells were treated for 72 h and subjected to SRB assay to determine cell viability and calculate IC50 values. IC50 values are shown in the inset tables. Cell viability is calculated as the percentage relative to the control treatment in each group (*n* = 3–4). (**B**) Dose-response curves for olaparib alone or in combination with a fixed concentration of 3 nM NOV202 in PC-3 and DU145 cells treated for 72 h. (**C**) Cell viability of PC-3 and DU145 cells treated for 72 h with olaparib (3 µM, 10 µM), NOV202 (3 nM), or their combinations. Cell viability is expressed as percentage of untreated control. Data are presented as mean ± SEM (*n* = 3). * *p* ≤ 0.05; ** *p* ≤ 0.01. (**D**) Combination index (CI) values for olaparib and NOV202 in PC-3 and DU-145 cells treated for 72 h. CI values were calculated using the Chou–Talalay method; CI < 1, CI = 1 and CI > 1 indicate synergy, additivity and antagonism, respectively, and the color scale denotes the degree of synergism or antagonism. The numerical CI value is shown in each cell. The black or white font of these values is used solely to maximize contrast against the cell color and carries no additional meaning

The antiproliferative effects of olaparib and NOV202 was investigated by SRB assay in the prostate cancer cell lines, PC-3 and DU145 in vitro, and IC50 values were calculated (Fig. 1A, C). Results showed different sensitivities of olaparib between the two cell lines. DU145 cells were 5 times more sensitive to olaparib (IC50 = 36.4 µM) than PC-3 cells (IC50 = 200 µM). In contrast, NOV202 was similarly effective in PC-3 cells (IC50 = 35.6 nM) and DU145 cells (IC50 = 29 nM) (Fig. 1A). Next, we examined whether NOV202 could further enhance the growth inhibitory response to olaparib. By adding NOV202 to olaparib, the growth inhibition was enhanced compared to either compound alone in both PC-3 and DU145 cells. Specifically, the addition of 3 nM NOV202 reduced the IC50 value of olaparib by 6.5-fold in PC-3 cells (from 200 µM to 31.5 µM) and by 3.6-fold in DU145 cells (from 36.4 µM to 10 µM) (Fig. 1B, C). Combination index (CI) analysis showed synergistic growth inhibitory effects at low NOV202 concentrations (3 and 15 nM) when combined with olaparib in both cell lines. (Fig. 1D).

### Olaparib induces DNA damage, apoptosis and ATM activation, which are not further enhanced by the combination

**Fig. 2:**
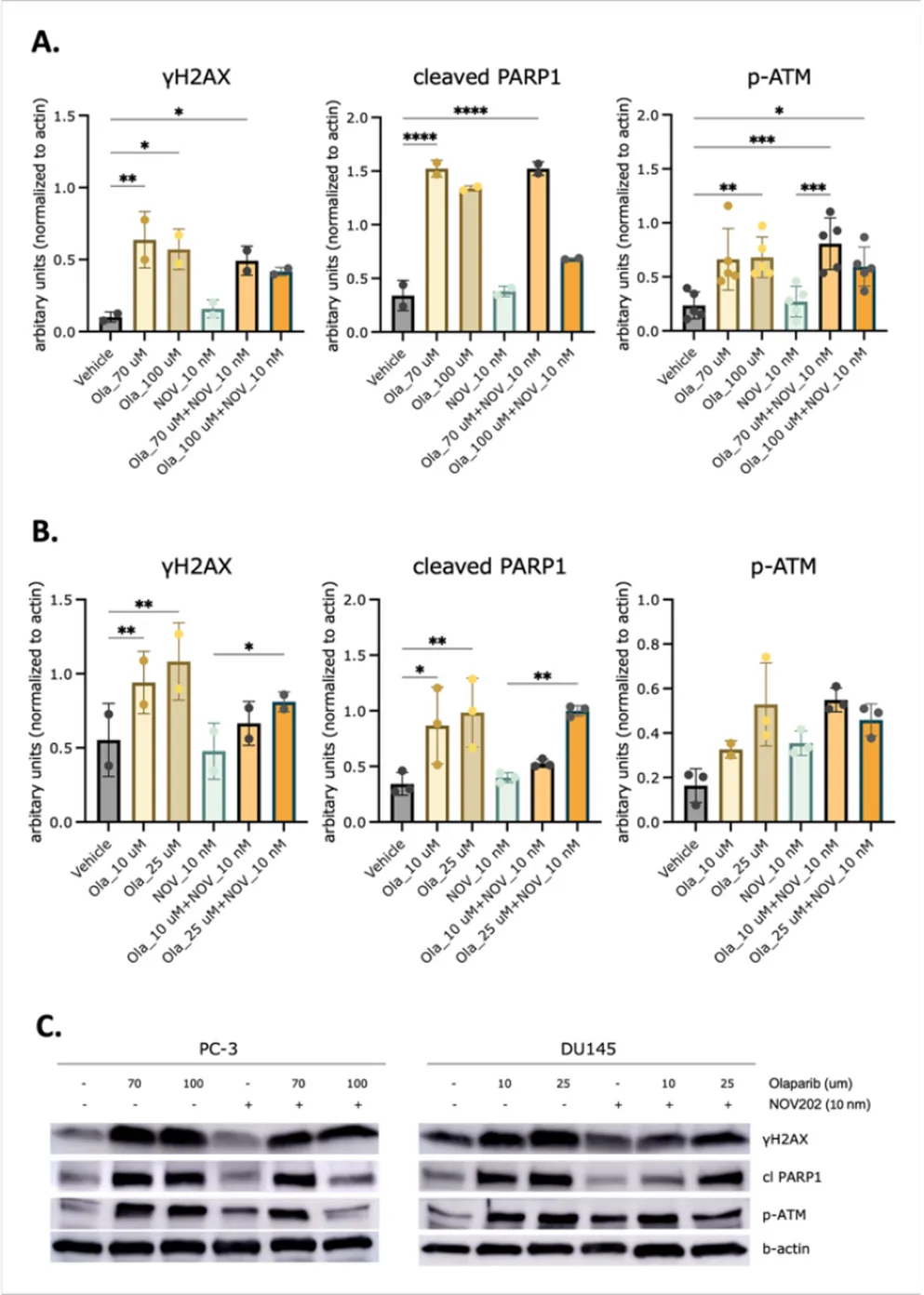
Expression levels of γH2AX, cleaved PARP1 and p-ATM in PC-3 (**A**) and DU145 (**B**) prostate cancer cells after treatment with olaparib (70 µΜ, 100 µΜ), NOV202 (10 nM) and their combinations. Cells were treated for 72 h under normoxia conditions (20% 0_2_). Protein expression levels were detected by western blot (**C**), with β-actin used as a loading control. Values represent mean ± SEM of at least three independent experiments. Statistical significance was determined using two-way ANOVA with the Tukey’s post hoc test; *p < 0.05; **p < 0.01; ***p < 0.001; ****p < 0.0001

We explored the effects of olaparib and NOV202, both as single agents and in combination, on the expression of DNA damage and apoptosis-related proteins in PC-3 and DU145 cell lines *in vitro.* Specifically, the levels of γH2AX, cleaved PARP, and phosphorylated ATM (p-ATM) were investigated (Fig. 2). Olaparib and the combination treatments both increased γH2AX and cleaved PARP1 levels in the two cell lines, indicating DNA damage and apoptosis. In PC-3 cells, p-ATM levels were significantly increased upon olaparib and combination treatment compared to NOV202 alone, suggesting activation of the ATM mediated DNA damage repair pathway, while these increases were moderate in DU145 cells. However, the combination treatment did not enhance the olaparib-induced effects at the concentrations used in this study.

### Olaparib and NOV202 inhibit tumor growth in prostate cancer xenografts

**Fig. 3:**
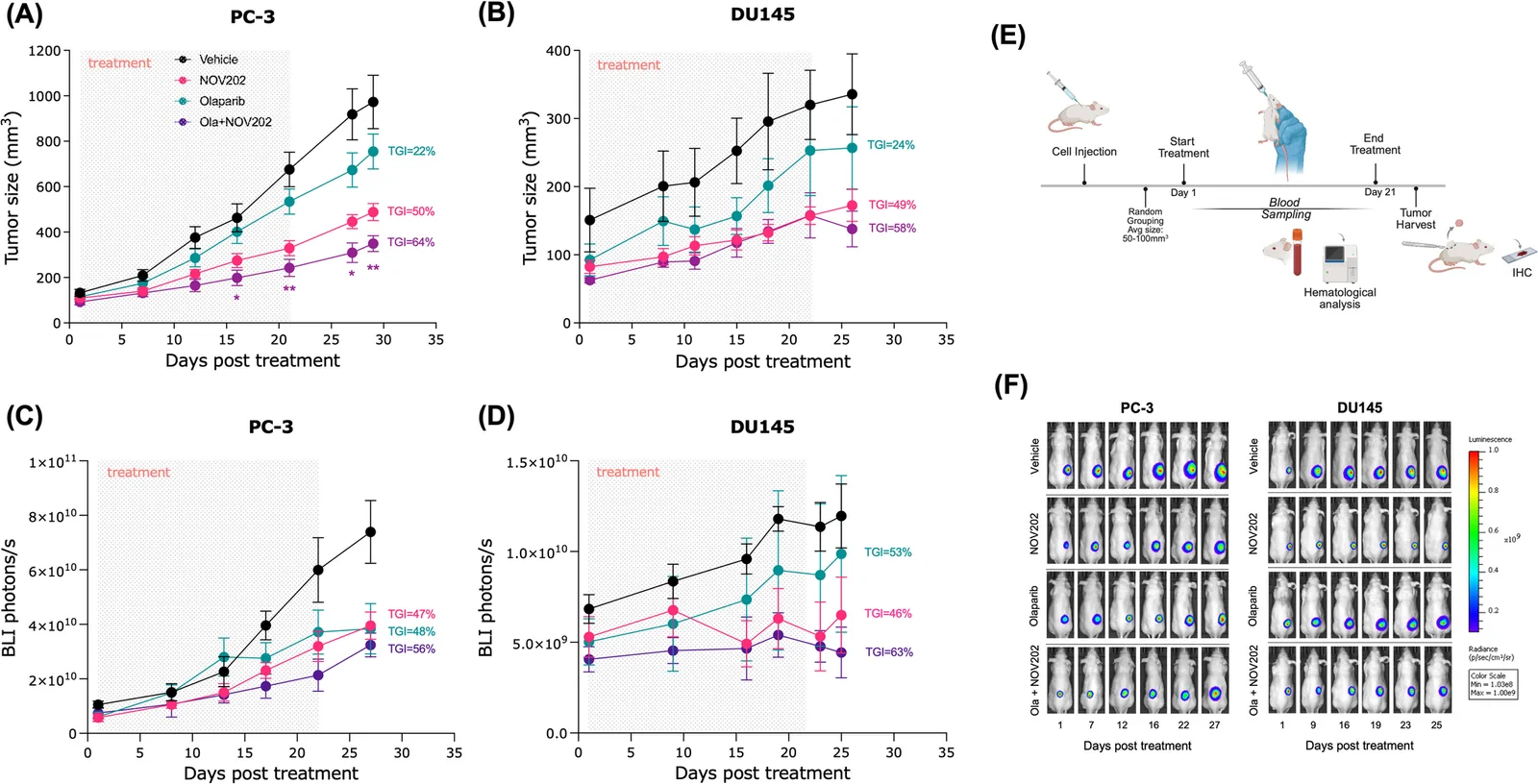
Tumor growth curves following treatment with vehicle, olaparib (100 mg/kg), NOV202 (30 mg/kg), or their combination are shown as caliper-measured tumor volume in PC-3 (**A**) and DU145 (**B**) xenografts, and as bioluminescence imaging (BLI) signal in PC-3 (**C**) and DU145 (**D**) xenografts. Panel (**E**) illustrates the in vivo experimental design, including tumor cell implantation, treatment timelines, blood sampling, and endpoint tissue collection for hematological analysis and immunohistochemistry. Representative BLI images acquired during the study are presented in panel (**F**). Total growth inhibition (TGI) values were calculated at the study endpoint. Data represent mean ± SEM; statistical analysis was performed using two-way ANOVA with Tukey’s post hoc test (*n* = 5–8 mice per group). Asterisks indicate significance versus the olaparib group: *p < 0.05, **p < 0.01

The antitumor effect of NOV202 and its potential to enhance the efficacy of the PARPi olaparib, were evaluated in PC-3 and DU145 prostate cancer xenografts. Selection of these two cell lines for the in vivo study was based on genetic profile analysis (Table 1, supplementary data), which shows that DU145 harbors mutations of pathogenic, likely pathogenic, or unknown significance in several genes, including a *BRCA2* mutation. In contrast, PC-3 lacks *BRCA2* mutations. Treatment with olaparib or NOV202 as single agents inhibited tumor growth in both xenograft models, compared to vehicle, as measured by caliper (Fig. 3A, B) and bioluminescence optical imaging (Fig. 3C, D). In PC-3 xenografts, NOV202 showed a stronger tumor growth-inhibitory effect than olaparib (TGI 50% and 22% respectively), and the combination gave a TGI of 64% (Fig. 3A). A similar trend was observed in DU145 tumors, where NOV202 and olaparib each reduced tumor growth (TGIs 49% and 24%, respectively), and the combination gave a TGI of 58% (Fig. 3B). Bioluminescence imaging (BLI) showed a similar pattern in both models (PC-3: TGIs 47% and 48% for monotherapies vs. 56% for the combination; DU145: 53% and 46% vs. 63%, respectively) (Fig. 3C, D). Overall, these data show that NOV202 has potent antitumor activity as a single agent in both prostate cancer xenograft models. Adding olaparib to NOV202 produced only a modest numerical increase in tumor growth inhibition, and the statistical comparisons shown in Fig. 3 refer to the olaparib monotherapy group; the present in vivo data therefore do not establish a robust combination benefit over NOV202 alone.

### Minimal preclinical toxicity signs associated with treatments in mice

Given clinical reports of olaparib inducing anemia, neutropenia and lymphopenia in clinical practice [26], we evaluated the in vivo preclinical toxicity of olaparib, NOV202 and their combination. Analysis shows lymphopenia and anemia by olaparib, NOV202 and combination treatment (Fig. 1 supplementary data). Based on weekly blood sampling, these changes were most pronounced during treatment and had recovered towards normal values by the study endpoint, indicating that they were transient over the tested dosing schedule. As no post-treatment follow-up was performed, these data support only short-term tolerability. Overall, the treatments were well-tolerated, showing only minor signs of hematological toxicity. Additionally, no significant loss in body weight was observed with the tested doses and administration schedule (Fig. 2 supplementary data).

### Olaparib and NOV202 increase DNA damage and reduce vascularization in prostate cancer xenografts

**Fig. 4:**
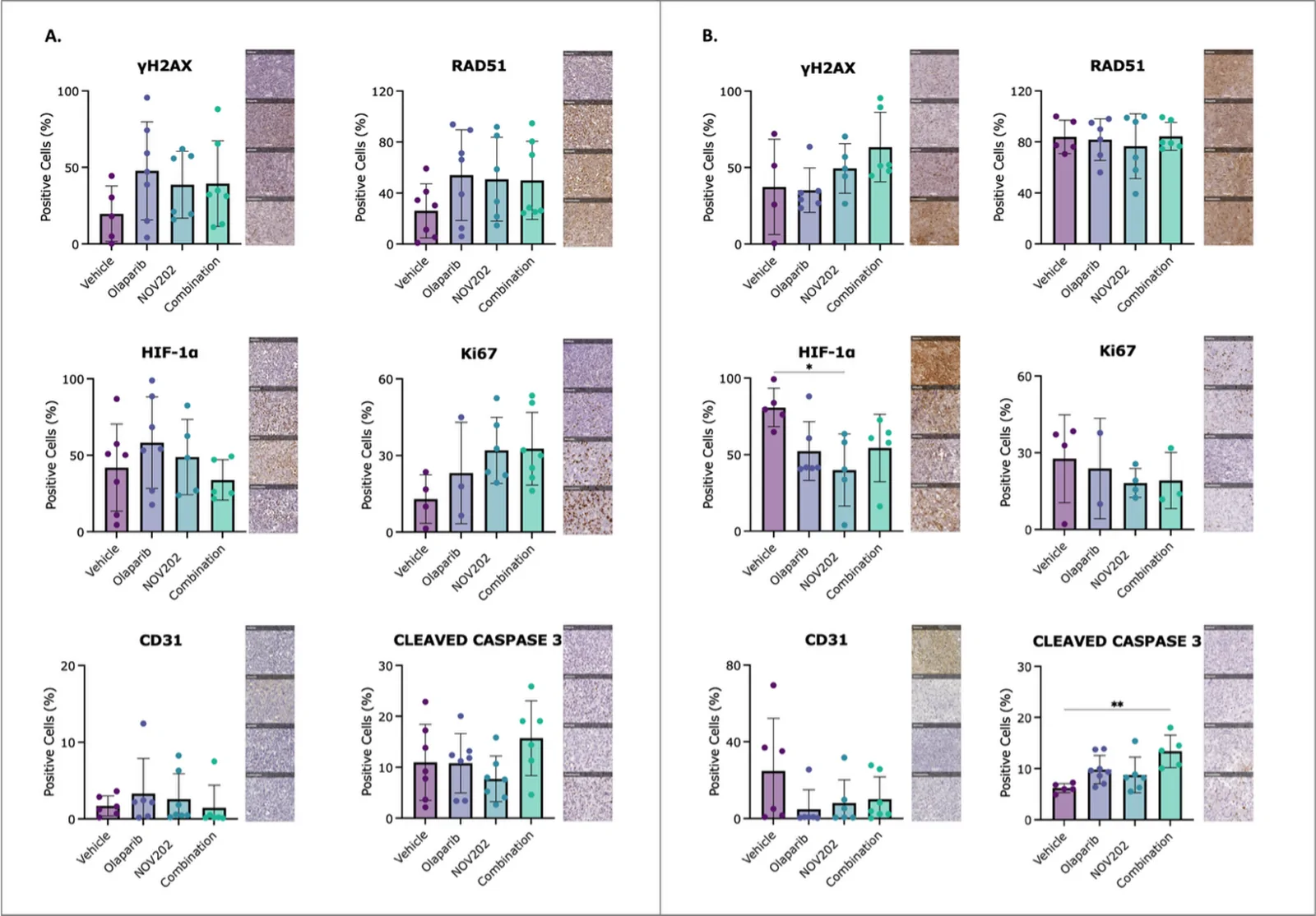
Immunohistochemical (IHC) expression of γH2AΧ, RAD51, HIF-1α, Ki67, CD31 and cleaved caspase 3 markers in PC-3 (**A**) and DU145 (**B**) xenografts. The results are presented as the mean ± SD; statistical significance was determined using one-way ANOVA with the Tukey’s post hoc test; *n* = 5–8 mice/group; *p < 0.05; **p < 0.01

To investigate treatment-associated cellular effects, primary tumors were immunostained for markers of DNA damage (γH2AX), homologous recombination repair (RAD51), hypoxia (HIF-1α), proliferation (Ki67), vascularization (CD31), and apoptosis (cleaved caspase-3) (Fig. 4). All treatments induced DNA damage, as evidenced by increased γH2AX levels. In PC-3 xenografts, RAD51 expression was upregulated by treatments, suggesting activation of homologous recombination repair pathways; however, RAD51 levels remained unchanged in DU145 xenografts. Reduced hypoxic response, by decreased HIF-1α levels was observed across all treatment groups in DU145. Ki67-positive cells increased by all treatments in PC-3 tumors but decreased in DU145, suggesting different effects on cellular proliferation. Additionally, CD31 expression decreased across all treatment groups, indicating reduced vascularization and the effect was more prominent in DU145. Cleaved caspase-3 levels were increased in the combination groups in both xenograft models, with a statistically significant increase observed in DU145 tumors compared with vehicle, consistent with increased apoptotic signaling. These immunohistochemical findings are reported as treatment-associated observations and do not establish the mechanism of the NOV202–olaparib interaction.

## Discussion

Metastatic castration-resistant prostate cancer remains a major clinical challenge due to tumor heterogeneity and frequent development of treatment resistance [1, 2]. PARP inhibitors have improved outcomes in patients with defects in HRR, particularly BRCA1/2 mutations [4–6] but responders with negative genetic testing have also been identified in clinical trials although the question of their applicability beyond HRR-mutated populations remains [17]. Currently, the presence of an HRR mutation is considered a prerequisite for PARPi use in mCRPC, sparking debate over whether HRR mutation is essential for PARPi response or if patients with proficient HRR can also benefit. Primary or acquired resistance to PARP inhibitors is still a clinical challenge. These limitations highlight the need for rational combination strategies capable of expanding PARPi applicability and overcoming resistance mechanisms.

Our study explored the therapeutic potential of combining PARP inhibitor olaparib with NOV202, a novel microtubule destabilizer and VDA, in prostate cancer xenograft models and in vitro settings.

In vitro, NOV202 showed potent antiproliferative activity with similar sensitivity across both PC-3 and DU145 cell lines. However, DU145 cells were more sensitive to olaparib than PC-3 cells, consistent with previously reported heterogeneity in PARP inhibitor responses among prostate cancer models. NOV202 significantly sensitized both cell lines to olaparib, reducing the olaparib IC50 by 6.5-fold in PC-3 and 3.6-fold in DU145 at low nanomolar concentrations. Combination index analysis confirmed synergistic interactions of the two compounds at nanomolar NOV202 doses suggesting that NOV202 enhances olaparib efficacy in vitro, resulting in synergistic growth inhibition.

Assessment of protein-level changes showed increased γH2AX and cleaved PARP1 levels after olaparib and combination treatment, consistent with treatment-associated DNA damage and apoptotic signaling. In PC-3 cells, increased levels of pATM, by olaparib and combination treatment, suggests activation of DNA damage repair processes. Given the lower sensitivity of PC-3 cells to olaparib, this ATM activation may reflect a compensatory repair response. Τhe combination did not further increase any of these DNA-damage or apoptosis markers beyond olaparib alone, and the mechanism underlying the interaction between the two compounds in vitro was not addressed in the present study.

In vivo, previous data indicated strong anticancer efficacy of NOV202 in ovarian cancer xenograft models. We aimed to determine if NOV202 could enhance olaparib’s anticancer efficacy in vivo. Our results showed that both olaparib and NOV202 as single agents significantly inhibited tumor growth in prostate cancer xenografts. NOV202 was the more active single agent in both models. The combination provided only a modest numerical improvement over NOV202 alone, and the statistically significant differences were observed relative to olaparib monotherapy. The in vivo data therefore demonstrate strong single-agent NOV202 activity rather than a robust additional benefit of adding olaparib. The synergistic interaction seen in vitro was observed at low NOV202 concentrations at which the compound alone was not maximally effective, whereas the dose used in vivo produced pronounced single-agent activity; the in vitro synergy therefore cannot be extrapolated to the in vivo setting. The reduction in tumor growth we observed was similar in both BRCA2 wildtype PC-3 xenografts and BRCA2-variant DU145 xenografts. However, it is important to note that the *BRCA2* mutation in DU145 cells is classified as a variant of unknown significance, not a confirmed pathogenic mutation. The absence of a BRCA2 knockout model restricted our ability to draw definitive conclusions regarding BRCA2-dependent responses. Antitumor activity was observed in both the BRCA2-wildtype PC-3 model and the DU145 model, which carries a BRCA2 variant of unknown significance. Because the study did not include a confirmed HR-deficient or isogenic BRCA2-null model, it does not establish whether the combination confers a selective advantage according to BRCA2 or homologous-recombination status.

Histological analyses provided additional context for the antitumor effects observed in vivo. All treatments increased γH2AX levels in both xenograft models, consistent with treatment-induced DNA damage and in agreement with the in vitro findings. Elevated RAD51 levels in PC-3 tumors may indicate that PC-3 cells can still use the HRR repair process to fix treatment induced DNA damage, which could be related to the increased proliferative activity reflected by Ki67 staining. In contrast, RAD51 levels did not increase after treatment in DU145 tumors, indicating that, unlike PC-3, these tumors did not upregulate RAD51 in response to treatment. The decrease in Ki67 expression indicates reduced proliferation in DU145 tumors. In DU145 tumors, the reduction in vascularization, as indicated by decreased CD31 levels, is consistent with the reported vascular-disrupting properties of NOV202. Cleaved caspase-3 was increased in the combination groups in both models, with a statistically significant rise in DU145 tumors, consistent with increased apoptotic signaling in the combination groups. These immunohistochemical analyses are descriptive and treatment-associated; they do not establish that DNA damage, impaired homologous-recombination repair, apoptosis or vascular disruption constitutes the mechanism of the NOV202–olaparib interaction, which would require dedicated mechanistic experiments.

Several limitations should be considered when interpreting these findings. The study was performed in two AR-null cell line xenograft models, PC-3 and DU145, which were selected to reflect the castration-resistant setting in which PARP inhibitors are used clinically. The in vitro interaction is reported without assigning it to BRCA2 or HRR status; determining whether the response depends on homologous-recombination status would require isogenic BRCA2-deficient and BRCA2-proficient systems or additional genetically defined models. In addition, the hematological changes observed here support only short-term tolerability, as no post-treatment follow-up was performed.

The preclinical nature of the study and its reliance on xenograft models, which may not fully represent the complexity of human mCRPC, further limit the conclusions that can be drawn. These findings support further preclinical investigation of the NOV202-olaparib combination, including studies to define its mechanism of action and to clarify its dependence on BRCA2 and homologous-recombination status.

In summary, NOV202 showed potent single-agent antitumor activity in both prostate cancer xenograft models and synergistic antiproliferative activity in combination with olaparib in vitro at low nanomolar concentrations. In vivo, the addition of olaparib did not provide a clear benefit beyond NOV202 alone, and the present data do not define the mechanism of the interaction between the two compounds or its dependence on homologous-recombination status. Within this defined preclinical scope, the study provides new information on the antitumor activity of NOV202 in prostate cancer models, while any benefit of combining it with PARP inhibition would need to be established in further preclinical studies.

## Supplementary Information

Below is the link to the electronic supplementary material.

Supplementary Material 1

## Data Availability

All data supporting the findings of this study are available within the paper and its Supplementary Material.
