# Supplementary material for: Combination of PARP inhibitor olaparib and a vascular disrupting agent reduces tumor growth in a preclinical model of prostate cancer
Source: Cancer Chemother Pharmacol. 2026 Sep 17;96(1):102. doi: 10.1007/s00280-026-04946-1 (PMC13586037; doi:10.1007/s00280-026-04946-1)
Supplement: Supplementary file 1 [file 280_2026_4946_MOESM1_ESM.docx]

**Table 1.** List of pathogenic, likely pathogenic, or unknown significance genes from the genetic profile analysis for PC-3 and DU145 cell lines.

| CHROM | CUR_HGV_C | CUR_HGV_P | MANUALLYCURATED | COMMENT | N_HET | N_HOM | N_GT | GENE | EXON | HGVS_C | HGVS_P | EFFECT | FUNCTION | SAMPLES |
| --- | --- | --- | --- | --- | --- | --- | --- | --- | --- | --- | --- | --- | --- | --- |
| 1 | c.1276C>T | p.(Arg426Cys) | 3 | true; ~99% alt allele | 3 | 0 | 19 | MUTYH | 13 | c.1276C>T | p.R426C | 0-Unknown | nonsynonymous_SNV | DU145-Luc2 |
| 2 | c.2206C>A | p.(Leu736Ile) | 3 | true; 30% alt allele | NA | NA | NA | MSH2 | 13 | c.2206C>A | p.L736I | 0-Unknown | nonsynonymous_SNV | DU145-Luc2 |
| 2 | c.3200G>T | p.(Ser1067Ile) | 3 | true; 68% alt allele | NA | NA | NA | MSH6 | 5 | c.3200G>T | p.S1067I | 0-Unknown | nonsynonymous_SNV | DU145-Luc2 |
| 3 | c.1757C>T | p.(Ala586Val) | 3 | true; 50% alt allele | NA | NA | NA | MLH1 | 16 | c.1757C>T | p.A586V | 0-Unknown | nonsynonymous_SNV | DU145-Luc2 |
| 7 | c.565C>T | p.(His189Tyr) | 3 | true; ~30% alt allele | NA | NA | NA | PMS2 | 6 | c.565C>T | p.H189Y | 0-Unknown | nonsynonymous_SNV | DU145-Luc2 |
| 13 | c.6851C>T | p.(Ser2284Leu) | 3 | true; ~100% alt allele | NA | NA | NA | BRCA2 | 12 | c.6851C>T | p.S2284L | 0-Unknown | nonsynonymous_SNV | DU145-Luc2 |
| 17 | c.395C>A | p.(Thr132Asn) | 3 | true; ~34% alt allele | 1 | 0 | 3 | BRIP1 | 5 | c.395C>A | p.T132N | 0-Unknown | nonsynonymous_SNV | DU145-Luc2 |
| 17 | c.668C>T | p.(Pro223Leu) | 3 | true; ~32% alt allele | NA | NA | NA | TP53 | 6 | c.668C>T | p.P223L | 0-Unknown | nonsynonymous_SNV | DU145-Luc2 |
| 17 | c.395C>A | p.(Thr132Asn) | 3 | true; ~34% alt allele | 1 | 0 | 3 | BRIP1 | 5 | c.395C>A | p.T132N | 0-Unknown | nonsynonymous_SNV | DU145-Luc2 |
| 17 | c.820G>T | p.(Val274Phe) | 4 | true; ~65% alt allele | NA | NA | NA | TP53 | 8 | c.820G>T | p.V274F | 0-Unknown | nonsynonymous_SNV | DU145-Luc2 |
| 3 | c.117-2A>T | NA | 5 | true; ~100% alt allele | NA | NA | NA | MLH1 | NA | c.117-2A>T |  | 0-Unknown | splice_acceptor | DU145-Luc2 |
| 16 | c.1320+1G>A | NA | 5 | true; ~30% alt allele | NA | NA | NA | CDH1 | NA | c.1320+1G>A |  | 0-Unknown | splice_donor | DU145-Luc2 |
| 17 | c.414del | p.(Lys139ArgfsTer31) | 5 | true; ~100% alt allele | NA | NA | NA | TP53 | 5 | c.414delC | p.A138fs | 0-Unknown | frameshift_deletion | PC3-Luc2 |
| 19 | c.532_536del | p.(Lys178GlyfsTer86) | 5 | true; ~100% alt allele | NA | NA | NA | STK11 | 4 | c.531_535del | p.I177fs | 0-Unknown | frameshift_deletion | DU145-Luc2 |

**Fig 1.** Blood parameter profiles of mice treated with NOV2020 (30mg/kg), olaparib (100mg/kg) and their combination for a 3-week treatment period in PC-3 (A) and DU145 (B) xenografts. Graphs show changes in the number of white blood cells (WBC), red blood cells (RBC), lymphocytes (LYM), monocytes (MON), neutrophils (NEU), hemoglobin concentration (HGB), hematocrit percentage (HCT), mean cell volume (MCV), mean cell hemoglobin (MCH), mean cell hemoglobin concentration, (MCHC), red cell distribution width (RDW), platelets count (PLT), mean platelet volume (MPV), platelet distribution width (PDW), procalcitonine test (PCT). In addition, the neutrophil to lymphocyte ratio (NLR) and the platelet to lymphocyte ratio (PLR) are calculated as inflammation markers. Results are presented as mean ± SEM.; n = 5-8 mice; statistical significance was determined using two-way ANOVA with the Tukey’s post hoc test; asterisks indicate significance versus vehicle group; ***p < 0.001; **p < 0.01; *p < 0.05.

**Figure 2.** Animal body weight relative changes after orally treatment for a 3-week treatment period and a 1-week post treatment period. Animals treated with NOV202 as monotherapy and in combination with olaparib experienced a 10-20% weight loss during treatment period followed by complete recovery until the end of the experiment.

**Fig.3** Immunohistochemical (IHC) representative images of γH2AΧ, RAD51, HIF-1α, Ki67, CD31 and cleaved caspase 3 markers in PC-3 (A) and DU145 (B) xenografts (enlarged from Figure 4 in the manuscript).
